# The Recurrence of Measles

**Published:** 1875-04-15

**Authors:** James I. Tucker

**Affiliations:** Chicago


### THE RECURRENCE OF MEASLES.

By James I. Tucker, A.M., M.D., Chicago.

THE epidemic of measles prevailing at the present time, of which Chicago comes in for her full share, affords an opportunity to observe the disease in many of its phases. Among these phases is its recurrency. It has been my privilege to attend a number of cases, and I have been surprised at the frequency with which I have been told (when, upon hearing the irritative cough, I have expressed my suspicions of measles) that this could not be the case, as the patient had already had them. But in every instance my suspicions have been confirmed. I make due allowance for the inaccuracy of the laity in matters of this kind, and recognize the possibility of mistakes on the part of medical men, but now and then a case occurs in the experience of individual practitioners, which so fully confirms the observation of others that it comes with the force of conviction.

Such a case is that of Carrie S——, aged ten years, whom I treated for measles in July, 1874. This was in every respect a typical case, in its prodromes, preliminary stage and exanthem. I am attending the same girl, who is now, March 23, 1875, abed with measles, this case differing from the other in no essential particular, the temperature in both cases rising to 103.2° F.

Instances of this kind are perhaps only worthy of mention, because the best authorities differ with regard to the possibility of a recurrence of measles. Even Rosenstein and Willan, who had the most ample opportunities, and devoted the closest attention to eruptive disease, state that they have never seen a case—at least one attended by fever. Probably second attacks are very rare, and differ in different epidemics, in this respect to be classified with variola, varicella, scarlatina, etc. Doubtless many cases are simply a re-appearance of the eruption after a temporary suppression. But if all cases were recorded, wherein occur two outbreaks in a single patient, at intervals of months or years, under the eye of a single practitioner, it might be found that such occurrences are more frequent than is generally supposed, and thus their authenticity established beyond a doubt.

Third attacks are probably very infrequent, but sometimes occur, as in Van Dievan's case (recorded Ziemssen, vol. 11, p. 43).

